# Outcome of Glansectomy and Skin Grafting in the Management of Penile Cancer

**DOI:** 10.1155/2011/240824

**Published:** 2011-04-04

**Authors:** Hugh F. O'Kane, Ajay Pahuja, K. J. Ho, Ali Thwaini, Thaigarajan Nambirajan, Patrick Keane

**Affiliations:** Department of Urology, Belfast City Hospital, Lisburn Road, Belfast BT9 7AB, UK

## Abstract

*Purpose*. To report outcome data for patients with penile cancer treated surgically with glansectomy and skin grafting. *Materials and Methods*. We retrospectively reviewed data on all patients undergoing surgical management of penile cancer by a single surgeon between 1998 and 2008. Outcomes in patients who underwent glansectomy and skin grafting were analysed. *Results*. Between 1998 and 2008 a total of 25 patients with a mean age 60 (39–83) underwent glansectomy and skin grafting. Six patients had carcinoma in situ (CIS); the stage in the remaining patients ranged from T1G1 to T3G3. Mean followup for patients was 28 months (range 6–66). Disease specific survival was 92% with 2 patients who had positive nodes at lymph node dissection developing groin recurrence. One patient developed a local recurrence requiring a partial penectomy. *Conclusions*. Penile preserving surgery with glansectomy and skin grafting is a successful technique with minimal complications for local control of penile carcinoma arising on the glans. Careful followup to exclude local recurrence is required.

## 1. Introduction

Penile cancer is an uncommon malignancy in the industrialized world, particularly in Europe and in the USA with an incidence of less than 1 per 100 000 of the male adult population. In contrast to this, the incidence in some parts of the developing world is as high as 19 per 100 000 per year [1].

More than 95% of penile cancers are primary squamous cell carcinomas with other uncommon histological types including melanoma, sarcoma, and basal cell carcinomas. Historically, the surgical management of the primary lesion in penile carcinoma has meant either partial or radical penectomy. Oncologically, radical surgical excision has stood the test of time, with excellent local control. These operations are however often mutilating and associated with urinary and sexual dysfunction as well as significant psychological morbidity [2].

In an attempt to reduce the negative impact of radical surgery and retain functional penile length, a variety of therapeutic strategies have been developed particularly for the management of more distal lower-grade cancers. The obvious risk is always that there will be compromise of local oncological control. Oncological outcomes of “penile preserving” surgical techniques should always, if possible, be measured against the gold standard of radical excision. Randomised trials are next to impossible in such an uncommon disease and although widely practiced and results are encouraging, only a small number of centres have published their outcome data on glansectomy and skin grafting for tumours involving the glans penis.

Here we report medium-term oncological and functional outcomes in a series of patients who underwent glansectomy and skin grafting for the treatment of distal penile carcinoma.

## 2. Materials and Methods

We reviewed data on all patients undergoing surgical management of penile cancer with glansectomy by a single surgeon at a tertiary referral centre between 1998 and 2008. All patients had biopsy proven squamous cell carcinoma or refractory carcinoma in situ and had their primary tumour clinically staged. Latterly, the majority of the patients underwent radiological staging with CT or MRI scan.

**Figure 1:**
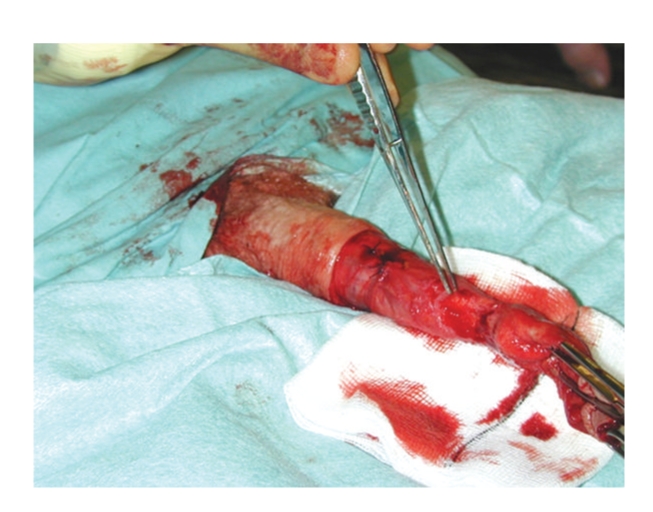
Under tourniquet control following an initial standard circumcision, the glans is detached with sharp dissection exposing the corporeal heads. The urethra is divided freeing the specimen.

**Figure 2:**
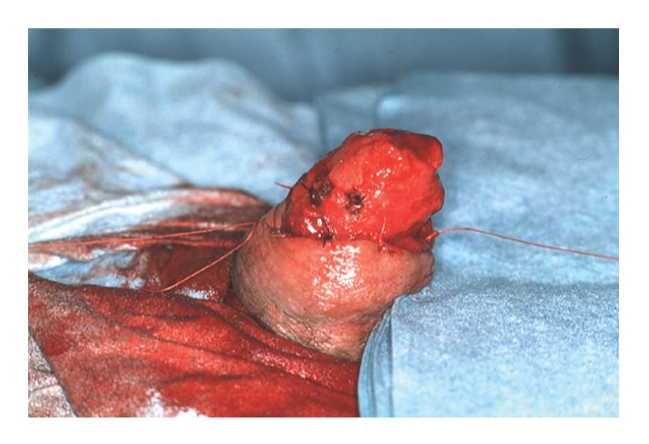
The penile skin is sutured proximally on to the corporal cavernosa leaving the corporal heads exposed for skin grafting.

**Figure 3:**
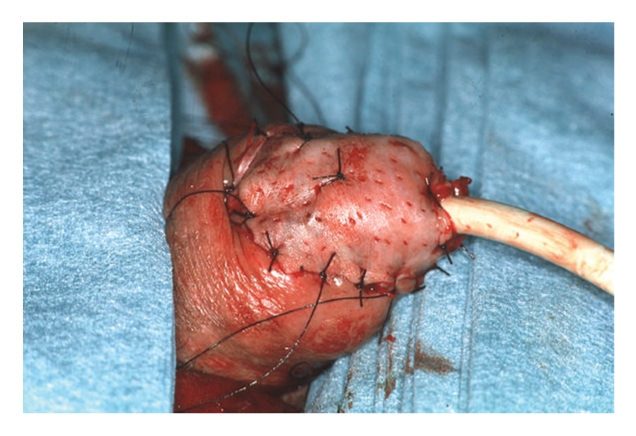
A partial thickness fenestrated skin graft harvested from the medial thigh is sutured with quilting sutures to the corporeal heads to form the neoglans.

Where suitable, glansectomy and split thickness skin grafting as described by Bracka was performed. The details of the surgical technique are outlined in Figures 1, 2, and 3. In summary, under tourniquet control following an initial standard circumcision the glans is detached with sharp dissection exposing the corporeal heads. The urethra is divided and spatulated freeing the specimen. The penile skin is sutured proximally on to the corporal cavernosa leaving the corporal heads exposed for skin grafting. A partial thickness fenestrated skin graft harvested from the medial thigh is sutured with quilting sutures to the corporeal heads to form the neoglans. Postoperatively the patient remains in bed with a catheter in situ for 5 days and the donor graft site managed in the standard fashion.

The regional nodes were managed dependent on the clinical and pathological staging of the primary tumour. Patients were followed up with regular review and clinical examination.

## 3. Results

Between 1998 and 2008, a total of 56 patients presenting with penile cancer underwent surgical treatment. Twenty five patients with a mean age of 60 (39–83) underwent penile preserving surgery with glansectomy and skin grafting performed by a single surgeon. Six out of 25 patients had CIS (carcinoma in situ). All the remaining patients had squamous carcinoma, the grade and stage of which are summarised in Table 1. Of the remaining 31 patients who underwent surgery, 26 had either partial or total penectomy with 4 managed by circumcision alone. Histology was squamous cell carcinoma in all but two patients who had melanoma.

Six of the 25 patients who were treated by glansectomy underwent bilateral modified groin node dissection including 2 patients with G3T1, two with G3T2, one with G3T3, and one patient with G2T1 disease. Of these patients, three (50%) demonstrated positive nodes. Mean followup for patients was 28 months (range 10–66). Disease-specific survival was 92% with 2 patients who had positive nodes at groin lymph node dissection developing groin recurrence. One patient who had G2T1 disease developed local recurrence requiring partial penectomy.

Nine out of 11 patients evaluated with regards to sexual function reported the ability to achieve erections. Six patients continued to be sexually active, one patient having fathered a child. There were no graft failures in our series. Two patients developed a meatal stenosis requiring dilatation; otherwise no other complications were noted.

## 4. Discussion

The treatment of uncommon malignancies such as penile cancer due to the difficulty in compiling good quality evidence-based treatment protocols means that the majority of the time treatment is based on historical strategies. Traditionally, the mainstays of treatment were either surgical amputation of part or all of the penis or radical radiotherapy. Surgical removal of a patient's penis often results in devastating anatomical, functional loss and a major psychological impact on the patient's life.

Eighty percent of penile carcinomas occur distally, involving the glans and/or prepuce and are potentially amenable to organ-preserving surgery. Innovative surgical techniques have focused on penile preservation in selected patients to minimize physical disfigurement and improve quality of life for these patients. Published series on the subject of penile conserving surgery has been limited to a small number of dedicated centres and leading experts in the field have encouraged reporting of results to encourage more widespread utilization of these techniques [3].

Very small lesions of the glans or prepuce may be suitable for local excision and primary closure or circumcision alone. Laser therapy with CO2 and neodymium:YAG based lasers have been used to either excise or ablate tumours or premalignant lesions such as carcinoma in situ. The results are satisfactory for premalignant lesions but recurrence rates of 17–33% have been reported in those patients with invasive tumours [4–6].

The main factor that perhaps has contributed to the move toward organ preserving surgery is the realization that traditional surgical margins of 2 cm are unnecessary to achieve good oncological results. In one series of 51 patients reported by Minhas et al., only 2 local recurrences were noted with surgical margins in this series, all less than 5 mm [7]. Surgical resection margin status has not been recorded in our series and is a recognised weakness; however, only one local recurrence in 25 patients has been noted at 28 months followup. Of note, frozen section at the time of surgery is now recommended with identified positive margins treated by wide local excision, a practice that we have now adopted.

The main types of penile preserving surgery that are currently in widespread use are partial or total glans resurfacing or glansectomy followed by skin grafting [8]. Other surgical techniques such as Mohs micrographic surgery (MMS), which involves removing the cancer by excising thin layers of tissue and examining them microscopically, have been described. Reported experience in this technique is very limited with only a small number of series published in the literature. It is a time-consuming procedure which appears frequently to require multiple treatments, and it is unclear whether it is a technique that is still practiced at this time [9, 10].

The early reporting of glansectomy on small numbers of patients demonstrated satisfactory outcomes with minimal complications and low recurrence rates in patients with malignant and nonmalignant conditions [11, 12]. The largest published series was a prospective study on 72 patients who underwent glansectomy with reconstruction for glans-confined penile squamous cell cancer. Local disease control was reported as excellent, with a 6% recurrence rate, despite 24 patients (33%) having high-grade tumours and 37 (51%) with T2 disease [13]. In our series, we have described 25 patients who underwent glansectomy and skin grafting with good cosmetic results and local control. Mean followup was 28 months with one patient developing local recurrence.

Although the retrospective nature of our study is a recognised weakness, all the procedures were performed by a single surgeon and satisfactory medium-term follow-up results have been achieved.

With regards to oncological control, penile preserving surgery compares favourably with results for radiotherapy, the other mainstay of penile cancer treatment that potentially allows for penile preservation. Although well-tolerated, recurrence rates of around 40% have been reported for external beam radiotherapy [14]. This compares unfavourably with the data from our series which had only 1 local recurrence (5%) which mirrors other larger reported series for penile preserving surgery which have demonstrated local recurrence rates of 2–4% [13, 15–17]. Brachytherapy would appear to achieve superior local control to external beam radiotherapy at 5 year followup achieving penile preservation in nearly 90% of patients. However, at 10 years this falls to 67% [18]. In the longer term meatal stenosis, urethral stricture disease and radiation necrosis are not infrequent complications of radiotherapy treatment which may necessitate more radical surgery [13]. Despite these problems radiotherapy remains an option for more proximal disease or in elderly patients unfit for anaesthesia.

Complication rates following penile preserving surgery with either resurfacing or glansectomy are extremely low. Reported rates of early graft loss following both resurfacing and glansectomy procedures requiring regrafting are low ranging from 3–10% [13, 17, 19]. None of the patients in our series required regrafting. One patient (5%) in our series suffered from meatal stenosis requiring self dilatation which mirrors the findings in some of the other reported series [13, 17]. Following grafting, the majority of authors advise strict patient immobilization to allow the skin graft to “take”. A potential improvement in the technique recently described involves leaving a proflavine-soaked gauze dressing “tied-over” the graft instead of using quilting sutures to fix the graft to corporeal heads. This has allowed almost immediate postoperative mobilization of the patient instead of the usual 4-5 days strict bed rest. In a reported series using this technique, only one patient out of 29 patients treated in this way required regrafting [19].

One of the main advantages of the penile preserving techniques is the potential for preservation of sexual function. One systematic review examining patient quality of life postsurgery for penile cancer demonstrated a negative effects on well-being in up to 40% with psychiatric symptoms in approximately 50% of patients. Up to two thirds of patients also reported a reduction in sexual function [2]. Another study demonstrated that, when asked, some men would even risk lower long-term survival to increase the chance of retaining sexual potency [20]. Preservation of sexual function following penile preserving surgery has been inconsistently reported in the published literature and none of the published series including ours have yet included validated tools or questionaires for evaluating sexual function. In two separate series of 17 patients treated by total or partial glansectomy with skin grafting, all patients were reported to maintain sexual function and activity postoperatively [15, 17]. In our series, only 11 patients were contactable to evaluate sexual function, 9 of whom could still achieve erections and 6 remaining sexually active. One explanation could be an older group of patients in our series (mean age 60 compared to 51 and 53). The prospective collection of data on sexual function pre- and postoperatively should be encouraged and is underway in a number of centres engaged in penile preserving surgery [21].

## 5. Conclusions

Penile amputation should be considered overtreatment in the vast majority of patients with penile cancer confined to the glans penis. Glans reconstruction with or without skin grafting has proven to be a successful technique with good follow-up data to support its use. Efforts to preserve penile length and function in the surgical treatment of penile cancer should be made in all suitable patients and there is good evidence to support this practice.

## Figures and Tables

**Table 1:** 

	G1 (%)	G2 (%)	G3 (%)
T1 (%)	6 (31%)	7 (37%)	2 (11)
T2 (%)	—	1 (5%)	2 (11%)
T3 (%)	—	—	1 (5%)
